# Maternal-Fetal Inflammation in the Placenta and the Developmental Origins of Health and Disease

**DOI:** 10.3389/fimmu.2020.531543

**Published:** 2020-11-13

**Authors:** Jeffery A. Goldstein, Kelly Gallagher, Celeste Beck, Rajesh Kumar, Alison D. Gernand

**Affiliations:** ^1^ Department of Pathology, Feinberg School of Medicine, Northwestern University, Chicago, IL, United States; ^2^ Department of Nutritional Sciences, College of Health and Human Development, Penn State University, University Park, PA, United States; ^3^ Section of Allergy and Immunology, Department of Pediatrics, Ann & Robert H. Lurie Children's Hospital and Northwestern University, Chicago, IL, United States

**Keywords:** maternal-fetal inflammation, placenta, DOHaD, chorioamnionitis, chronic villitis, asthma, neurodevelopmental outcomes

## Abstract

Events in fetal life impact long-term health outcomes. The placenta is the first organ to form and is the site of juxtaposition between the maternal and fetal circulations. Most diseases of pregnancy are caused by, impact, or are reflected in the placenta. The purpose of this review is to describe the main inflammatory processes in the placenta, discuss their immunology, and relate their short- and long-term disease associations. Acute placental inflammation (API), including maternal and fetal inflammatory responses corresponds to the clinical diagnosis of chorioamnionitis and is associated with respiratory and neurodevelopmental diseases. The chronic placental inflammatory pathologies (CPI), include chronic villitis of unknown etiology, chronic deciduitis, chronic chorionitis, eosinophilic T-cell vasculitis, and chronic histiocytic intervillositis. These diseases are less-well studied, but have complex immunology and show mechanistic impacts on the fetal immune system. Overall, much work remains to be done in describing the long-term impacts of placental inflammation on offspring health.

## Introduction

The developmental origins of health and disease (DOHaD) theory, in which *in utero* or early life events can have a significant impact on adult outcomes, has become the organizing principle of fetal and perinatal biology (1–3). Extensive research has focused on maternal nutritional status and later metabolic disease in offspring, but some of the most striking DOHaD findings come from examination of the long term impact of exposure to inflammation. *In utero* exposure to the 1918 (Spanish) influenza pandemic has been associated with increased hospitalizations, heart disease, and cancer in middle age and older survivors (4, 5). In the last decade, the placenta has become a new focus within DOHaD research (6). A recent paper described the placenta as the “center of the chronic disease universe” (7). While the U-shaped relationship between birthweight and risk of heart disease has been reported across numerous studies and populations, less recognized is the similar U-shaped relationship between the ratio of placental weight to birthweight and coronary heart disease (8, 9). Placental inflammation is a sub-focus in the study of chronic disease risk, particularly within the context of the global obesity epidemic and low-level, chronic inflammation that is present in pregnant women with a high BMI. Rigorous characterization of inflammation in the placenta is a longstanding component of pathological examination, yet diagnoses are complex and poorly understood outside of perinatal pathology (10). The purpose of this review is to first examine the inflammatory lesions in the placenta and describe their characteristics. For each lesion, we then describe the associations with long-term outcomes and relate studies relevant to potential or known mechanisms.

## Acute Placental Inflammation (API)

Acute placental inflammation (API) is the microscopic equivalent to the clinical diagnosis of chorioamnionitis (11, 12). The term histologic chorioamnionitis has been used and is still used as a stage of maternal inflammatory response (which is a subcategory of API, discussed below). The difference in terminology reflects that, while API is strongly associated with clinical chorioamnionitis, it can be seen without symptoms and signs of clinical chorioamnionitis (13, 14). Significantly, low-stage API can be seen in up to 50% of uncomplicated vaginal deliveries following uncomplicated pregnancies (15).

### API, Acute Inflammation and Infection

The relationship between API and other forms of inflammation and infection is complex, hence the retirement of prior terminology including amniotic fluid infection (AFI), intrauterine infection (IUI), and ascending infection (11). Presumed pathogenic bacteria are identified in 72% (16), 89% (17), 38% (18), 61% (14), and 4% (19) of cases, depending on the clinical circumstances and methodology. In general, bacteria are more frequently identified in preterm delivery and when API and clinical chorioamnionitis are present. Distinguishing sterile API vs. API with bacterial contaminants vs. API with bacterial bystanders vs. API with *bona fide* pathogenic bacteria is challenging and likely blurs our understanding of the epidemiology and long-term consequences of this lesion. For example, in a study of amniotic fluid collected before rupture of membranes, women with elevated IL-6 were likely to deliver preterm regardless of culture or PCR results (20). Does this indicate that sterile inflammation is real and problematic, or that the microbiologic results are false negatives?

If acute inflammation is not in response to infection, what is the stimulus? *In vitro* studies suggest the forces of labor themselves induce inflammation. Mechanical stretch induces expression of cyclooxygenase 2 (COX2), activator protein 1 (AP1), NF-κB, and connexin 43 in amnion explants (21, 22). Mechanical stretch of immortalized human myometrial cells induced expression of multiple cytokines, including IL-6 and IL-12, chemokines CXCL8 and CXCL1, and induced transendothelial migration (23). These studies support a path from sporadic contractions (i.e. Braxton-Hicks) or labor to acute inflammation. Further, maternal obesity causes low-grade inflammation that may be reflected in the placenta and associated adverse pregnancy outcomes (24, 25).

#### Maternal Inflammatory Response (MIR)

**Figure 1:**
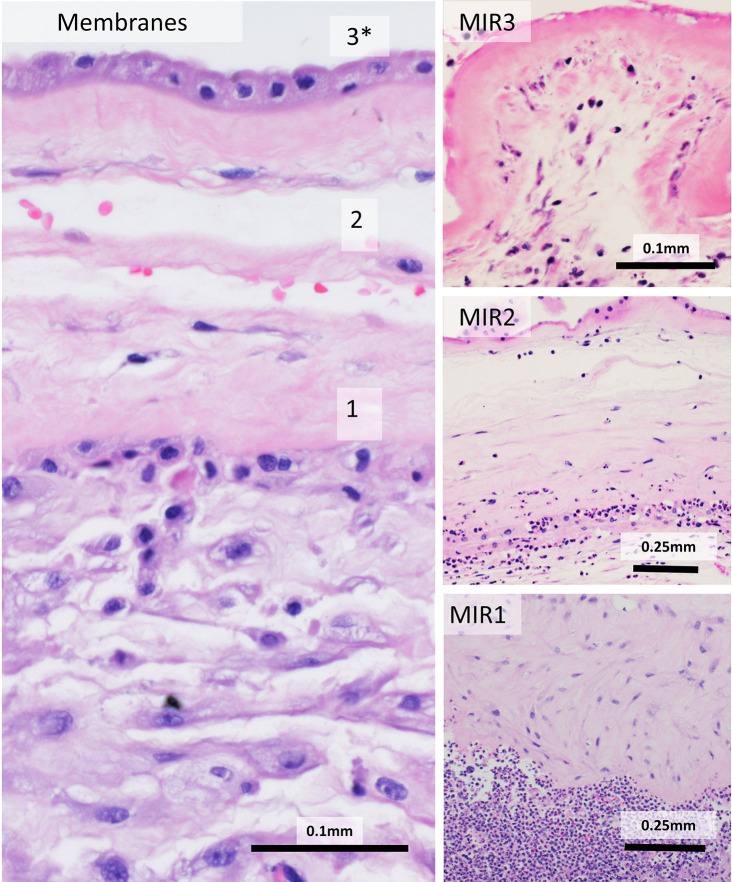
Maternal inflammatory response (MIR) stages: Normal membranes (left) contain amnion (top), fibrous chorion (middle) and decidua (bottom). Maternal inflammation is staged by the location and state of neutrophils. Neutrophils lined up at the decidua/chorion border are MIR1. Once neutrophils cross into the chorion, MIR2 is reached. Neutrophilic debris, death of amnion cells, and thickened basement membrane are diagnostic of MIR3.

API is divided into the **maternal** **inflammatory response** (MIR) and **fetal inflammatory response** (FIR) depending on the source of the inflammatory response (26). MIR is staged 1 to 3, with higher stages corresponding to a longer exposure to insult. Histologically, MIR consists of extravasating maternal neutrophils which approach and then cross into the chorionic layer, move through the amnion and into the amniotic space (**Figure 1**). MIR is staged as **subchorionitis** (Stage 1) when neutrophils congregate at the border between the subchorionic fibrin and chorion in the chorionic plate or between the cellular and fibrous chorion in the extraplacental membranes. Inflammation of the chorion (**chorionitis**) or chorion and amnion (**chorioamnionitis**) is Stage 2 - the gap between chorion and amnion not acting as a significant barrier to the passage of neutrophils. MIR Stage 3, so called **chorioamnionitis with amnion necrosis,** can be diagnosed on the basis of amniocyte necrosis, but is more reliably diagnosed by the presence of neutrophil karyorrhectic debris (11, 12). Based on rhesus models, analogy, and expert experience, Stage 1 MIR tends to occur 6 to 12 h after exposure to an inflammatory stimulus, Stage 2 MIR occurs at 12 to 36 h, and Stage 3 MIR indicates exposure of >36 h (27).

#### Fetal Inflammatory Response (FIR)

**Figure 2:**
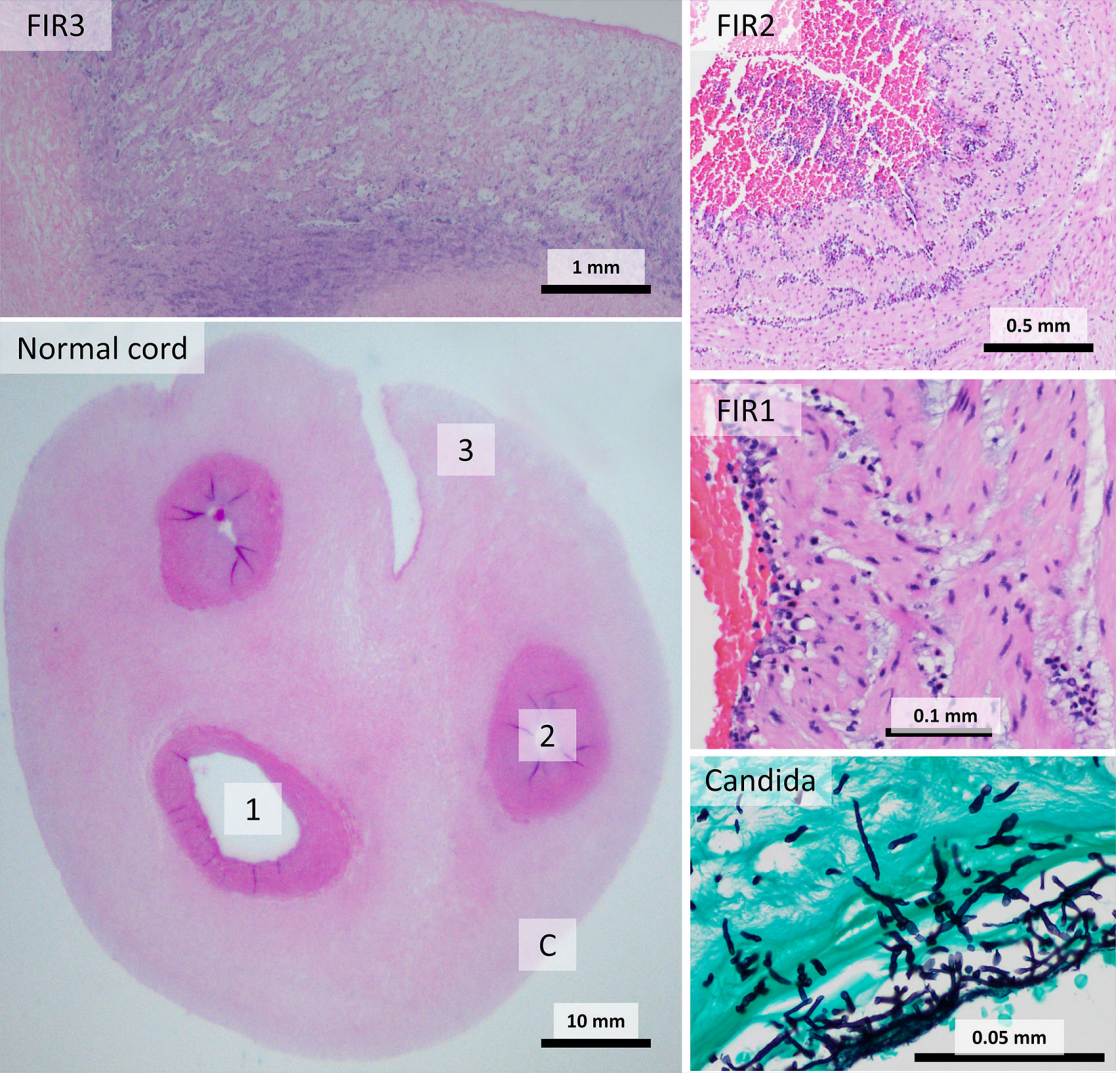
Fetal inflammatory response (FIR) stages: A normal umbilical cord (bottom left) includes two arteries (circular vessels) and one vein (larger, ovoid vessel) surrounded by Wharton’s jelly. FIR1 consists of inflammation of the vein. Arterial inflammation is diagnostic of FIR2. Inflammation of Wharton’s jelly with necrosis is FIR3. *Candida* infections produce peripheral abscesses with invasive organisms (Grocott Methenamine Silver stain).

FIR consists of extravasating fetal neutrophils, which traverse fetal tissues to and move toward the amniotic space (**Figure 2**). FIR is at Stage 1 when neutrophils are seen crossing fetal vessels in the chorionic plate (**chorionic vasculitis**) or involving the umbilical vein (**phlebitis**). Inflammation of the umbilical arteries (**arteritis**) indicates Stage 2, while inflammation of Wharton’s jelly with necrosis, **necrotizing funisitis** is Stage 3. In contrast, non-necrotizing funisitis is ambiguous. In the clinical literature, funisitis is used to mean any FIR in the umbilical cord. In the pathologic literature, funisitis is defined as neutrophilic infiltration of Wharton’s jelly, any degree of which was considered diagnostic of FIR stage 2. The significance of this finding has been down-graded in the pathology literature (11). Timing of FIR lesions is less clear than MIR, possibly reflecting the differing maturation of the fetal immune system over the course of gestation.

#### Acute Villitis

**Figure 3:**
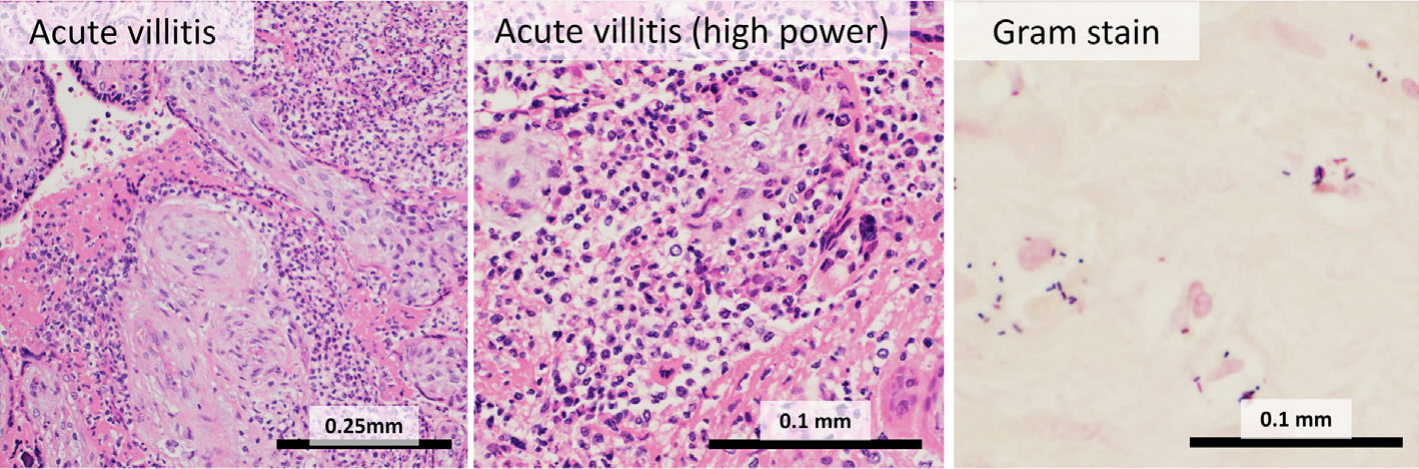
Acute villitis: In acute villitis, terminal villi show dense inflammation with fibrin. Gram stain demonstrates bacterial forms.

Acute villitis is an uncommon histological pattern which involves neutrophil infiltration of the chorionic villi beneath the trophoblastic membrane, and can occur with or without chorioamnionitis (28) (**Figure 3**). Acute villitis is associated with maternal sepsis from listeriosis, and with other infections, usually bacterial, including Group B *Streptococci, Klebsiella, Escherichia coli, Campylobacter, Haemophilus*, tuberculosis and syphilis (12). Acute villitis is suggestive of acute fetal infection with serious fetal consequences, including fetal sepsis and death (29).

#### Immunology of API

Neutrophils are the major cell type involved in API (11, 30). Outside the placenta, maternal leukocytosis is one of the criteria for clinical chorioamnionitis, while fetal complete blood count shows leukocytosis and neutrophilia (31, 32). Monocyte/macrophages are increased in amniotic fluid in clinical chorioamnionitis, indicating they also undergo migration through the placenta in response to inflammatory stimuli (33). Bacterial products, such as lipopolysaccharide (LPS) would be expected to induce a classical activation pattern (termed M1) in macrophages (34). Perhaps surprisingly resident maternal decidual macrophages, showed a switch toward an alternatively activated (M2) polarization in API, while fetal macrophages resident in the terminal villi were primarily M2 at baseline, which was unaffected by clinical chorioamnionitis (35, 36). Clinical chorioamnionitis is associated with a change in umbilical cord monocyte histone marks, suggesting reprogramming of the fetal immune system as well (37). M1/M2 (and M2-subtype) polarization has been more extensively studied in animal models compared to humans (38).

Eosinophils are an uncommon component of the acute inflammatory response in general, but are frequently encountered in the fetal inflammatory response of preterm infants (39, 40). This is presumed to be due to the immaturity of the fetal immune system. The significance of eosinophil predominant versus neutrophil predominant fetal inflammatory response has been incompletely explored. However the synergy between preterm delivery and chorioamnionitis as risk factors for asthma and wheezing (explored in more detail below) is suggestive (41).

Alterations in fetal and neonatal T-cells have been identified. Within the umbilical cord, high stage API was associated with an increased proportion of Foxp3+ cells coexpressing retinoic acid receptor-related orphan receptor gamma T (RORγT) (42). Preterm API was also associated with a shift toward the T helper 17 (Th17) phenotype, including increased numbers of progenitor and mature Th17 cells, IL-17+ Treg cells and effector memory T-cells that coexpressed Th17 antigens (43). Fetal tissues also showed altered lymphocytes. In stillbirths complicated by API, splenic Foxp3+ cells were decreased, while pulmonary CD3+ cells were increased (42).

The humoral components of clinical chorioamnionitis and API are well-studied (26). Umbilical cord and post-delivery infant blood show increased levels of IL-1, IL2R, IL-6, IL-8, TNF-α, MIP-1β, RANTES, and I-TAC (44–47). The fetus may respond directly to bacteria that enter either through the bloodstream (sepsis) or through inhalation of bacteria-laden amniotic fluid (pneumonia).

#### Hereditary Risk of API

A genome wide association study (GWAS) of clinical chorioamnionitis using DNA from newborn blood spots showed no genome-wide associations. However, several exonic variants in inflammation-associated genes showed nominal significance, including Fc receptor like 5 (*FCRL5*), interleukin 23 receptor (*IL23R*), phospholipase A2 receptor 1 (*PLA2R1*), complement C1 receptor (*C1R*), interleukin 10 receptor alpha (*IL10RA*), DNA cross-link repair 1C (*DCLRE1C*), TRAF3 interacting protein 1 (*TRAF3IP1*), and fibroblast growth factor 3 (*FGFR3*) (48). Variants in *TRAF3IP1* and *FGFR3* have been associated with changes in the forced expiratory volume in 1 s over forced vital capacity ratio (FEV1/FVC), a diagnostic feature of asthma (49, 50). Several genes show associations with a variety of infectious and autoimmune conditions. *IL23R* variants are associated with autoinflammatory conditions, including inflammatory bowel disease, psoriatic arthritis, and autoimmune conditions in pediatric patients (51, 52). *PLA2R1* variants are associated with autoimmune membranous glomerulonephritis and inflammatory bowel disease (53, 54). *IL10RA* variants have been associated with pneumonia in adults (55). Interestingly, *DCLRE1C* variants have been associated with response to cognitive behavioral therapy in anxiety and migraine gesturing toward neurocognitive outcomes (56, 57).

A study on placental (fetal) genotype from API cases, also focusing on immune-associated genes, found an association between chorioamnionitis, a promoter variant in interleukin 6 (*IL6*), methylation of the *IL6* promoter and *IL6* gene expression (58). Significantly *IL6* variants have been associated with asthma and childhood onset of asthma (59).

#### Clinical Associations With API

##### Neonatal Mortality and Morbidity

Maternal inflammatory response is associated with adverse neonatal outcomes when combined with fetal inflammatory response (60, 61) and fetal inflammatory response alone is often associated with poor outcomes (62–65). Multiple studies demonstrate an increased risk of neonatal death in the presence of FIR (60, 63, 66). Early onset sepsis is associated with FIR (62, 63) as are severe retinopathy of prematurity (61) and necrotizing enterocolitis and spontaneous intestinal perforation in the preterm (64).

##### Respiratory Outcomes

Bronchopulmonary dysplasia (BPD) is the most common respiratory disorder in preterm infants characterized by an interruption in pulmonary vascular and alveolar development which may originate in the antepartum, intrapartum or postpartum period (67). The role of placental inflammation and BPD is conflicting, with some studies finding an association between FIRS and BPD (63, 68) and histological chorioamnionitis and BPD (69) while other studies find either no association between placental inflammation and BPD (65) or a decreased risk of BPD with histological chorioamnionitis with fetal inflammatory response (70).

In preterm infants, API or MIR2 are risk factors for recurrent wheeze (71), asthma (41, 72), and chronic lung disease (73) but not altered lung function (71, 74). Preterm birth is an independent risk factor for both API and respiratory disease. (26, 75, 76). A series of studies from overlapping groups of authors have used causal path analysis to untangle this interdependency (73, 77, 78). In one study, MIR and FIR were directly causative of chronic lung disease of prematurity and indirectly causative through their influence on prematurity and mechanical ventilation (77). A more recent study re-demonstrated a direct effect of FIR on chronic lung disease of prematurity, which then had a risk of progression to asthma in childhood (73). These studies are valuable but include relatively few patients and are sensitive to permutations in model design.

The mechanism of the inflammation-lung outcomes association in animal chorioamnionitis models has been suggested to be related to altered metalloproteinase activation in the airway (79–81) and FOXP3 CNS 3 methylation, decreasing the balance of Treg and Th17 cells (82, 83). However, these studies used an acute endotoxin injection model which is more in keeping with API. Stillborn fetuses and liveborn infants exposed prenatally to API and chronic villitis both had Treg and Th17 marker co-expressing cells (42) which may suggest a shift in Tregs to a Th17 phenotype (43, 84). Another study also showed elevated numbers of Th17 cells in cord blood of only very preterm neonates, with a trend to lower Tregs/Th17 ratios in preterm infants who were exposed to chorioamnionitis. This same study also showed a trend to higher numbers of Tregs co-expressing the canonical IL-17 transcription factor RORγt, again suggesting a shift to Th17 type immunity in the context of histological chorioamnionitis (43). One reason these immune deviation effects are seen more in preterm infants is that there is a developmental shift to Th17 cells in preterm children (85). RNA sequencing of cord blood from a small number of infants suggests that there may be additional pathways such as changes in CCR2 and other pathways involved in T cell survival and Treg development (86). These shifts in Th17 and Treg patterns may have implications on Th2/Th17 high endotypes of asthma (87–89).

##### Neurocognitive and Developmental Outcomes

Intraventricular hemorrhage and periventricular leukomalacia are serious complications in preterm neonates. Intraventricular hemorrhage in preterm is the most common cause of hydrocephalus and increases the risk for poor neurodevelopment outcomes (90). Periventricular leukomalacia is a type of preterm brain injury associated with adverse neurodevelopment (90), including cerebral palsy (91, 92). Both intraventricular hemorrhage and periventricular leukomalacia are associated with FIRS (62, 63, 65).

The association between API and neurocognitive outcomes has been extensively examined with mixed results. As with asthma, the three-way association between prematurity, API, and adverse outcomes raises issues of causation. Using data from the Collaborative Perinatal Project (1959–1976), Liu et al. showed an association between FIR and low IQ scores (93). Specifically, FIR in early preterm infants (20–34 weeks) was associated with an increased risk of low IQ (<70) at 4 years, but not 7 years. FIR in term infants was associated with an increased risk of low Performance IQ (vs. Verbal IQ or Full Spectrum IQ) at 7 years, but not 4 years. These findings are compelling, but the use of multiple subgroups and measures, and the lack of consistency between ages 4 and 7 years complicate interpretation. In a recent meta-analysis of studies using the Bayley II developmental scale, MIR was associated with a lower mental development index, but a nonsignificant *increase* in the motor development index (94). In a case-control study of 254 children, API was associated with an increased risk of autism spectrum disorder, with a further elevated risk in FIR (95). Further complicating matters is the possible interaction of histological chorioamnionitis and clinical chorioamnionitis. An observational study of the Eunice Kennedy Shriver National Institute of Child Health and Human Development National Research Network with 2,390 extremely preterm infants found that histological chorioamnionitis alone when adjusted for gestational age was associated with lower odds of poor neurodevelopment outcomes whereas histological combined with clinical chorioamnionitis resulted in an increased risk of cognitive impairment at 18 to 22 months when compared to no chorioamnionitis (96).

Many studies have challenged this association. A study of 86 infants born prematurely in Orlando, Florida showed no difference in Bayley scale at 1 year when infants were matched for gestational age, birth weight, respiratory distress syndrome, and intraventricular hemorrhage grade (97). In a study from Western Australia, MIR2 in preterm infants born <30 weeks gestation was not associated with decreased Bayley III developmental scores at 2 years (98). At three years, a study of 2,201 children born <34 weeks gestation from Japan showed no difference in cerebral palsy, risk of developmental quotient <70, or neurodevelopmental impairment between pregnancies with and without MIR2 (99). A matched-case control study of extremely preterm infants with CP examined the role of placental pathology and found no association between histological chorioamnionitis or funisitis and CP (100).

The mechanism by which API may cause neurologic impairment is unclear. In rodents, maternal injection with lipopolysaccharide, a Gram-negative bacterial component, induces neurocognitive and behavioral abnormalities without fetal infection (101). In a small case-control study, severe FIR and severe MIR were associated with cerebral palsy (CP) in very low birthweight infants (<1,500 g) (102). However, the relationships were indirect. Using a series of logistic regressions, severe FIR was associated with CP via its association with thrombi in fetal vessels, while severe MIR was associated with CP via its association with villous edema. Thromboemboli (from FIR) or under perfusion (from MIR) would then be the immediate cause of CP. A study from Sweden using Bayley-III scales and developmental outcomes at an adjusted 2.5 years of age to diagnose CP in extremely preterm children suggests placental infarction as a contributor to CP but did not find associations with other placental pathology outcomes (103).

#### API Associated With *Candida*

Infection by *Candida albicans* results in a distinct pathologic appearance, most characteristically punctate abscesses on the periphery of the umbilical cord (peripheral funisitis,) (30, 104). In preterm infants, *Candida* is associated with cutaneous candidiasis, sepsis, pneumonia and a high rate of perinatal death, while at term it is more often an incidental finding (104–106). Foreign bodies, such as retained intrauterine device or uterine cerclage are risk factors for *Candida* (104, 107). *Candida glabrata* and *Candida lusitania* are associated with *in vitro fertilization* and are associated with high risk of adverse outcomes. API due to *Candida* is relatively rare. The immunologic features and long-term consequences are unknown.

## Chronic Placental Inflammation (CPI)

**Figure 4:**
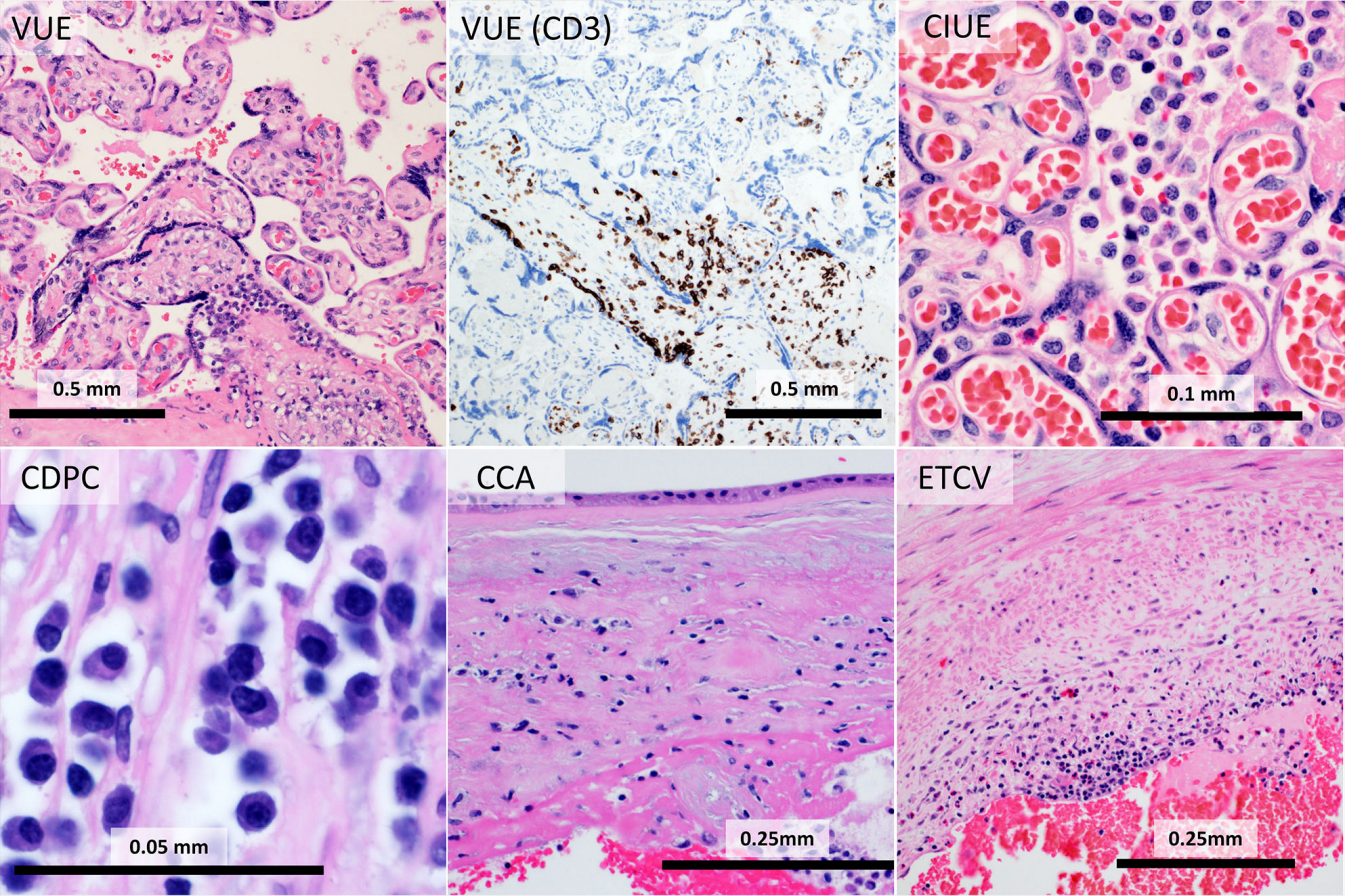
Chronic placental inflammation (CPI): In chronic villitis of unknown etiology (VUE), CD3-positive T-cells infiltrate fetal villi. Chronic intervillositis of unknown etiology (CIUE) is characterized by intense histiocytic inflammation filling the intervillous space. Chronic deciduitis with plasma cells (CDPC) shows plasmacytic inflammation in the decidua. Chronic chorionitis (CCA) consists of maternal T-cells in the chorion. In eosinophilic T-cell vasculitis (ETCV), fetal T-cells and eosinophils inflame fetal vessels.

The chronic inflammatory lesions of the placenta are a group of frequently co-occurring lymphocytic, histiocytic, and plasmacytic processes distinguished by the cells present and their location in the placenta (108) (**Figure 4**). Diseases with high rates of maternal-fetal transfer, including *Toxoplasma, Treponema*, rubella, cytomegalovirus (CMV), herpesvirus (HSV1 and HSV2), human immunodeficiency virus (HIV1, “TORCH” infections), are the most commonly identified in CPI (109). The effect of congenital TORCH infections has been extensively reviewed elsewhere. Therefore, this review will focus on the >95% of cases in which no etiology is identified (110). Two competing theories have arisen to explain these cases 1) That CPI results from failure of maternal tolerance to fetal antigens or 2) That unknown or untested-for infectious agents in the placenta induce a maternal response, akin to transplant rejection (111). Evidence for the alloimmune theory includes the increased frequency of CPI in egg donor pregnancies, where the fetus is fully allogeneic, rather than ½ self and ½ allogeneic (112, 113). Conversely, interbreeding of inbred mouse strains is associated with immune activation and resorption, the degree of which is strain dependent (114). Activation of the maternal immune system by lipopolysaccharide (LPS), a Gram negative bacterial component, or polyinosine:cytosine (poly-IC), a viral mimetic, increases the rate of resorption, prompting a model of immune activation in an allogeneic background (115, 116).

### Chronic Villitis (VUE)

Chronic villitis of unknown etiology (VUE) is a process involving infiltration of placental villi by lymphocytes, histiocytes, and rarely plasma cells (117). VUE most commonly affects terminal villi, the sites of gas and nutrient exchange, closest to the maternal surface (“basal villitis”), however it frequently is present in other sites and, more rarely, is diffuse (12). In addition to lymphocytic infiltration, VUE is characterized by aggregation of terminal villi, destruction of villous capillaries (resulting in “avascular villi”) and stem villous vessels (“stem villous obliteration”). The antigen is unclear, however the destruction of endothelium and sparing of syncytiotrophoblast is suggestive. The prevalence of VUE in studies is estimated at 5% to 15% of placentas submitted for pathologic examination (118). However, the diagnosis is frequently missed by inexperienced or nonspecialized pathologists, doubtless impairing research (119, 120). The lymphocytes in chronic villitis are maternal in origin based on human leukocyte antigen (HLA) mismatch (121). The lymphocytes are primarily CD8, but include T-regulatory (Treg) cells and retinoic acid receptor-related orphan receptor gamma (ROR**γ**T) cells (42, 122). The mechanism by which T-cells pass the maternal-fetal barrier is unknown. Lymphocytes and histiocytes in VUE express inflammatory cell adhesion molecule ICAM1, supporting a model similar to typical leukocyte extravasation (108). Alternatively, maternal inflammatory cells may pass through disruption of the trophoblastic barrier. Lymphocytes and histiocytes also show expression of nuclear factor kappa B (NFκB) (108). Histiocytes express HLA-DR and histiocytes and syncytiotrophoblast show phosphorylated Signal Transducer and Activator of Transcription 1 (STAT1), indicating activation of the JAK-STAT pathway.

Chronic villitis is associated with increased maternal and fetal plasma chemokines CXCL9, CXCL10, and CXCL11 (123). Within the placenta, there is increased expression of chemokine mRNAs for CXCL9, CXCL10, CXCL11, CXCL13, CCL4, and CCL5 and chemokine receptor mRNA for CXCR3 and CCR5 (123). Chronic villitis is also associated with deposition of the complement component C4d on villous surfaces or fetal vascular endothelium, indicating a potential role for complement-mediated processes (124).

It is unclear whether maternal cells in VUE fully cross from the placenta to seed the developing fetal immune system. T-cells of presumed maternal origin have been identified in cord blood, children and adults based on XY fluorescence in-situ hybridization (FISH) or HLA testing (125, 126). In cord blood, the rate of this maternal microchimerism has been reported at 23% (127). The significance of these maternal cells and their interaction with autoimmune conditions is complicated (125). For example, maternal T-cells are more common in blood from patients with juvenile dermatomyositis (an autoimmune condition) than those with muscular dystrophy (a genetic condition) (128). However, in biopsies of injured muscle, the situation is reversed and there are more maternal T-cells in muscular dystrophy (129). Unfortunately, studies on T-cells of maternal origin have not cross-referenced examination of the placenta.

### Chronic Deciduitis With Plasma Cells (CDPC)

This lesion is characterized by a lymphocyte and plasma cell infiltrate in the decidua (12, 130). CDPC is histologically similar to chronic endometritis - plasmacytic inflammation of the uterus in the absence of pregnancy (110). The antigen is unknown, but major histocompatibility complex/human leukocyte antigen mismatch appears to play a role (131). Maternal immunoglobulin gamma (IgG) is actively transported across the placenta, representing a straightforward mechanism for effects on the fetus (132, 133). Transplacental passage of IgG is the mechanism for hemolytic disease of the newborn, neonatal alloimmune thrombocytopenia, and congenital heart block among others (134–136). However, these diseases are not characterized by CDPC, raising the question of whether the plasma cells in CDPC are acting in a paracrine fashion.

### Chronic Chorionitis/Chronic Chorioamnionitis (CCA)

Chronic chorionitis (CCA) is defined by lymphocytic or lymphoplasmacytic infiltration of the chorion or chorion and amnion (117). CCA is frequently associated with VUE (137, 138). The lymphocytes are primarily CD8 T-cells, with few CD4; B-cells and NK-cells are uncommon (138). Amniotic fluid concentrations of the chemokines CXCL9 and CXCL10, along with their receptor, CXCR3, are elevated in CCA (139). CXCL9, -10, and -11 mRNA are upregulated in placental membranes with CCA (137).

### Chronic Intervillositis of Unknown Etiology (CIUE)

Chronic intervillositis is an uncommon condition in which maternal histiocytes and to a lesser extent lymphocytes fill the intervillous space (140, 141). As in other CPI conditions, it can be seen in association with infectious causes particularly malaria and cytomegalovirus, however this review will focus on the idiopathic chronic intervillositis of unknown etiology (CIUE) (142, 143). CIUE may occur in association with chronic villitis, or as a purely isolated finding. Controlled trials have not been performed, but the successful treatments support an alloimmune or prothrombotic mechanism for CIUE (144).

In CIUE, the intervillous histiocytes are M2-polarized with overexpression of complement receptor 4 (CD11c/CD18) and toll-like receptor 1 (TLR1) (142, 143, 145, 146). Unlike VUE, the T-cells in CIUE are a mixture of CD4 and CD8-cells, with admixed Tregs (147).

### Eosinophilic T-Cell Vasculitis (ETCV)

Eosinophilic T-cell vasculitis (ETCV) is an uncommon chronic inflammatory condition first described in 2002 with an incidence of 0.2 to 0.7% of pregnancies (111). It consists of fetal eosinophils, histiocytes, and T-cells present in the wall and lumen of large fetal vessels (111, 148). It most commonly presents with involvement of a single chorionic plate vessel, often with an associated thrombus (149). ETCV occurs more often than chance with VUE of thrombotic conditions (149, 150). However, ETCV is frequently an isolated finding, reinforcing its place as an independent diagnosis. ETCV is differentiated from API by the absence of maternal inflammation, orientation of inflammatory cells toward the placental disc rather than toward the amnion, and the different inflammatory populations.

The infiltrate in ETCV is poorly characterized, however some facts are known. In contrast to VUE, the T-cells of ETCV are of fetal origin (148). The T-cells are a mixture of CD25+, FOXP3+ Tregs and other T cells (150). Long term outcomes have not been well described, likely related to the low incidence, recent description, and frequent co-occurrence of other pathologies.

### Clinical Associations With CPI

Relative to API, fewer studies have examined CPI. Outcomes sometimes associated with CPI include: pregnancy loss; preterm delivery; growth restriction; a possible association with neonatal alloimmune thrombocytopenia; and neurocognitive and developmental outcomes. Additionally, the risk of recurrence is high with many chronic inflammatory lesions.

#### Pregnancy Loss

Villitis of unknown etiology is associated with fetal death and recurrent loss (110, 117, 151). In one study focused on stillbirth, placentas with VUE were analyzed and it was found that a Th1-type immune response predominated (151). Fetal demise is seen in chronic deciduitis with plasma cells though fewer studies have evaluated this pathology (151). Chronic chorioamnionitis is also associated with fetal death (117, 152). Chronic intervillositis of unknown etiology is a strongly associated with miscarriage, intrauterine fetal demise and a very high risk of recurrence (141, 153–155). Women with a history of recurrent CIUE have gone on to successful live birth after treatment with aspirin and low molecular weight heparin (LMWH), aspirin and corticosteroids, aspirin, LMWH, and steroids, or aspirin, prednisone, LMWH and hydroxychloroquine (147, 156, 157).

#### Preterm Delivery

Although VUE is sometimes associated with preterm labor (117), chronic chorioamnionitis is most frequently associated with late spontaneous preterm birth (117, 158). A study of 1206 preterm births found that chronic chorioamnionitis was most frequently associated with late preterm birth designated as 34 to 37 weeks while acute chorioamnionitis was most commonly associated with very early preterm birth designated as less than 28 weeks (158). Chronic deciduitis with plasma cells is also associated with preterm labor, but has not been established as an independent risk factor for long-term outcomes (158, 159).

#### Growth Restriction

Villitis of unknown etiology is associated with fetal growth restriction, low birth weight and small for gestational age (110, 117, 160). Chronic intervillositis of unknown etiology is the other chronic inflammatory pathology frequently associated with disorders of fetal growth (155, 161–163). Multiple studies demonstrate outcomes of fetal growth restriction with rates of 70% or higher when CIUE is present (155, 161, 162).

#### Neonatal Alloimmune Thrombocytopenia

Neonatal alloimmune thrombocytopenia (NAIT) is a rare pregnancy complication characterized by otherwise unexplained severe thrombocytopenia in a neonate (164). Analogous with immune hydrops, NAIT is caused by maternal alloimmunization against fetal antigens. An association between VUE and neonatal alloimmune thrombocytopenia (NAIT) has been reported (165). This study examined histopathology from 14 placentas of pregnancies affected by NAIT and found that chronic villitis was observed in untreated pregnancies compared with intravenous immunoglobulin treated pregnancies. This one small study links the histological observation of VUE to placentas affected by NAIT. As NAIT is driven by alloimmunization, the association with VUE provides further evidence that VUE is an alloimmune process.

#### Neurocognitive and Developmental Outcomes

Among patients with intrauterine growth restriction, VUE was associated with an increased risk of low developmental index at 2 years of age (166). In another study, VUE with stem villous obliteration was associated with an increased risk of cerebral palsy or other abnormal neurodevelopmental findings in term infants (167). The limitation to VUE with stem villous obliteration in this work was for comparison to other conditions causing stem villous anomalies and there is no evidence to suggest VUE without stem villous obliteration will have a different impact. Another study with term infants with hypoxic-ischemic encephalopathy found that chronic villitis was associated with injury in the basal ganglia and thalamus (168). Chronic chorioamnionitis has also been associated with white matter injury in newborns, but this increased risk was seen in newborns with chronic chorioamnionitis and funisitis while neither condition alone was associated with white matter injury (169). This study suggests that the interaction of insults rather than one clear etiology may be responsible for initial neurocognitive insults.

## Null Associations

Many studies examining associations between inflammatory lesions and specific short- and long-term outcomes have not found meaningful relationships. Long term outcomes of isolated CCA have not been described. n a systematic review of associations with stillbirth, neonatal morbidity, and neurologic outcomes, null findings formed the bulk of those reported (159).

## Conclusion

**Figure 5:**
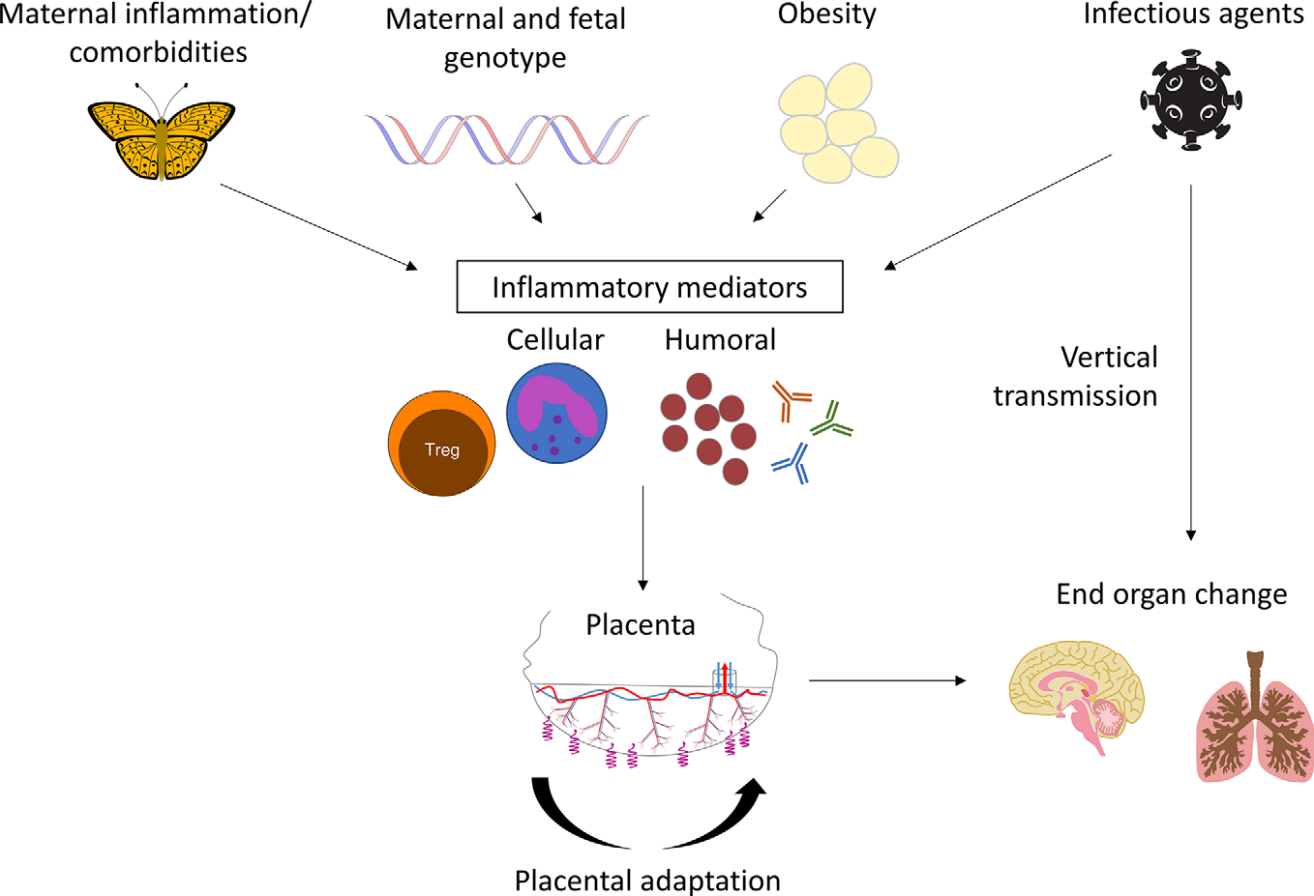
Model of placental inflammation in pregnancy: Maternal inflammation integrates maternal autoimmune diseases, genetic risk factors, obesity, and infectious organisms. Cellular and humoral mediators induce placental adaptation and maladaptation. The final results are lifelong abnormalities in end organ function.

Maternal-fetal inflammation frequently involves the placenta, broadly grouped into API and CPI. Each has numerous subtypes and degrees of inflammation. Both present an inflammatory shock to the fetus, driven by maladaptation in the placenta and have been associated with long-term adverse outcomes, including asthma, cerebral palsy, abnormal neurodevelopment, and autism spectrum disorder (**Figure 5**). Other than the classical API response to presumed ascending infection, the long-term outcomes of these diseases are poorly studied and additional associations are likely to be identified with focused research. Potential differences in outcomes by placental/fetal sex are also needed. While the NIH Human Placenta Project was established to drive discoveries in real-time placental function in utero, there has been an overall recognition of how little we know about the placenta’s relationship to the health of humans. Additional studies of placental pathology, particularly inflammatory lesions, could contribute greatly to the DOHaD field.

## Author Contributions

All authors contributed to the article and approved the submitted version. JG created the microphotographs. **Figure 5** was drafted by CB.

## Funding

JG is supported by NIBIB K08 EB030120.

## Conflict of Interest

The authors declare that the research was conducted in the absence of any commercial or financial relationships that could be construed as a potential conflict of interest.
